# Supplementary material for: The effects of genital myiasis on the diversity of the vaginal microbiota in female Bactrian camels
Source: BMC Vet Res. 2022 Mar 5;18:87. doi: 10.1186/s12917-022-03189-5 (PMC8897907; doi:10.1186/s12917-022-03189-5)
Supplement: Supplementary file 5 — Additional file 5. [file 12917_2022_3189_MOESM5_ESM.zip › MPL201709200_16s_yy/Treat1/css/js_css/pdfmake-master/examples/pdfs/named_styles.pdf]

This is a header, using header style
Lorem ipsum dolor sit amet, consectetur adipisicing elit. Confectum ponit legam, perferendis nomine miserum, animi. Moveat nesciunt triari naturam.

Subheader 1 - using subheader style
Lorem ipsum dolor sit amet, consectetur adipisicing elit. Confectum ponit legam, perferendis nomine miserum, animi. Moveat nesciunt triari naturam posset, eveniunt specie deorsus efficiat sermone instituendarum fuisse veniat, eademque mutat debeo. Delectet plerique protervi diogenem dixerit logikh levius probabo adipiscuntur afficitur, factis magistra inprobitatem aliquo andriam obiecta, religionis, imitarentur studiis quam, clamat intereant vulgo admonitionem operis iudex stabilitas vacillare scriptum nixam, reperiri inveniri maestitiam istius eaque dissentias idcirco gravis, refert suscipiet recte sapiens oportet ipsam terentianus, perpauca sedatio aliena video.

Lorem ipsum dolor sit amet, consectetur adipisicing elit. Confectum ponit legam, perferendis nomine miserum, animi. Moveat nesciunt triari naturam posset, eveniunt specie deorsus efficiat sermone instituendarum fuisse veniat, eademque mutat debeo. Delectet plerique protervi diogenem dixerit logikh levius probabo adipiscuntur afficitur, factis magistra inprobitatem aliquo andriam obiecta, religionis, imitarentur studiis quam, clamat intereant vulgo admonitionem operis iudex stabilitas vacillare scriptum nixam, reperiri inveniri maestitiam istius eaque dissentias idcirco gravis, refert suscipiet recte sapiens oportet ipsam terentianus, perpauca sedatio aliena video.

Lorem ipsum dolor sit amet, consectetur adipisicing elit. Confectum ponit legam, perferendis nomine miserum, animi. Moveat nesciunt triari naturam posset, eveniunt specie deorsus efficiat sermone instituendarum fuisse veniat, eademque mutat debeo. Delectet plerique protervi diogenem dixerit logikh levius probabo adipiscuntur afficitur, factis magistra inprobitatem aliquo andriam obiecta, religionis, imitarentur studiis quam, clamat intereant vulgo admonitionem operis iudex stabilitas vacillare scriptum nixam, reperiri inveniri maestitiam istius eaque dissentias idcirco gravis, refert suscipiet recte sapiens oportet ipsam terentianus, perpauca sedatio aliena video.

Subheader 2 - using subheader style
Lorem ipsum dolor sit amet, consectetur adipisicing elit. Confectum ponit legam, perferendis nomine miserum, animi. Moveat nesciunt triari naturam posset, eveniunt specie deorsus efficiat sermone instituendarum fuisse veniat, eademque mutat debeo. Delectet plerique protervi diogenem dixerit logikh levius probabo adipiscuntur afficitur, factis magistra inprobitatem aliquo andriam obiecta, religionis, imitarentur studiis quam, clamat intereant vulgo admonitionem operis iudex stabilitas vacillare scriptum nixam, reperiri inveniri maestitiam istius eaque dissentias idcirco gravis, refert suscipiet recte sapiens oportet ipsam terentianus, perpauca sedatio aliena video.

Lorem ipsum dolor sit amet, consectetur adipisicing elit. Confectum ponit legam, perferendis nomine miserum, animi. Moveat nesciunt triari naturam posset, eveniunt specie deorsus efficiat sermone instituendarum fuisse veniat, eademque mutat debeo. Delectet plerique protervi diogenem dixerit logikh levius probabo adipiscuntur afficitur, factis magistra inprobitatem aliquo andriam obiecta, religionis, imitarentur studiis quam, clamat intereant vulgo admonitionem operis iudex stabilitas vacillare scriptum nixam, reperiri inveniri maestitiam istius eaque dissentias idcirco gravis, refert suscipiet recte sapiens oportet ipsam terentianus, perpauca sedatio aliena video.

It is possible to apply multiple styles, by passing an array. This paragraph uses two styles: quote and small. When multiple styles are provided, they are evaluated in the specified order which is important in case they define the same properties
